# Gene expression changes by high-polyphenols cocoa powder intake: a randomized crossover clinical study

**DOI:** 10.1007/s00394-018-1736-8

**Published:** 2018-06-08

**Authors:** P. K. Barrera-Reyes, N. Hernández-Ramírez, J. Cortés, L. Poquet, K. Redeuil, C. Rangel-Escareño, M. Kussmann, I. Silva-Zolezzi, M. E. Tejero

**Affiliations:** 1 Nutrigenomics and Nutrigenetics, National Institute of Genomic Medicine, 14610 Mexico City, Mexico; 2 Vitamins and Phytonutrients, Nestlé Research Centre, 1000 Lausanne, Switzerland; 3 Computational Genomics, National Institute of Genomic Medicine, 14610 Mexico City, Mexico; 4 Systems Nutrition, Metabonomics and Proteomics, Nestlé Institute of Health Sciences, 1015 Lausanne, Switzerland; 5 Present Address: Liggins Institute, 1142 Auckland, New Zealand; 6 Metabolic Programming, Nestlé Research Centre, 1000 Lausanne, Switzerland

**Keywords:** (−)-Epicatechin, Molecular, Antioxidant, Flavanol, PBMCs, Microarray, ROS, Catechin

## Abstract

**Purpose:**

To assess the effect of the intake of a single dose of high-polyphenols cocoa on gene expression in peripheral mononuclear cells (PBMCs), and analyze conjugated (−)-epicatechin metabolites in plasma, which may be related with an antioxidant response in healthy human.

**Methods:**

A randomized, controlled, double-blind, cross-over, clinical trial in healthy young adults who consumed a single dose of high-polyphenols cocoa powder and maltodextrins as control, with a one-week washout period. Analysis of circulating metabolites, plasma antioxidant capacity and gene expression changes in PBMCs were performed under fasting conditions and 2-h after treatment using microarray in a subsample. Pathway analysis was conducted using Ingenuity Pathway Analysis (IPA).

**Results:**

Twenty healthy participants (9 F) were included in the study. A significant increase in circulating (−)-epicatechin metabolites was found after cocoa intake in all participants without related changes in antioxidant capacity of plasma. The metabolites profile slightly varied across subjects. Treatments triggered different transcriptional changes in PBMC. A group of 98 genes showed changes in expression after cocoa treatment, while only 18 were modified by control. Differentially expressed genes included inflammatory cytokines and other molecules involved in redox balance. Gene and network analysis after cocoa intake converged in functions annotated as decreased production of reactive oxygen species (*p* = 9.58E−04), decreased leukocyte activation (*p* = 4E−03) and calcium mobilization (*p* = 2.51E–05).

**Conclusions:**

No association was found between conjugated metabolites in plasma and antioxidant capacity. Changes in PBMCs gene expression suggest anti-inflammatory effects.

**Electronic supplementary material:**

The online version of this article (10.1007/s00394-018-1736-8) contains supplementary material, which is available to authorized users.

## Introduction

Polyphenols are a large family of plant-derived molecules generally involved in defense against ultraviolet radiation or aggression by pathogens. Polyphenols from cocoa, mainly flavanols, may affect multiple risk factors for chronic diseases in human, including higher blood pressure, dyslipidemia, inflammation, insulin resistance, vascular reactivity and other oxidative stress-related diseases [1–4]. Health benefits of cocoa polyphenols are widely documented in epidemiological and experimental studies. Inferences about their capacity to protect cell constituents against oxidative damage through free radicals scavenging has been accepted for the last years [5–9]. However, this concept has been recently challenged due to recent updates on the pharmacokinetics and physiology of cocoa polyphenols, which questioned their antioxidant capacity [10, 11]. As proposed by Scalbert et al. [6], the effect of polyphenols is more likely mediated by direct interactions with surface membranal receptors involved in signal transduction, which results in a modulated redox status of the cell and may induce a series of redox-dependent reactions. In this context, a number of studies in cell culture have demonstrated that cocoa polyphenols are capable to modulate intracellular calcium and prevent oxidation by down-regulation of inflammatory mediators [tumor necrosis factor alpha (TNFα), enzymes cyclo-oxygenase-2 (COX-2), inducible nitric oxide synthase (iNOS), nuclear factor kappa-light-chain-enhancer of activated B cells (NF-kB), activator protein-1 (AP-1), cytokines and nuclear transcription factor erythroid 2p45-related factor [Nrf2] in Jurkat T cells, HepG2 cells and Caco-2 cells [12–15]. *Ex vivo* studies using circulating human cells and animal models have found a reduced expression of biomarkers of endothelial dysfunction and inflammation [interleukins 1 beta (IL-1β), interleukin 2 (IL-2), interleukin 4 (IL-4)], interleukin 6 (IL-6), E-selectin and vascular cell adhesion molecule (VCAM)-1) [16–19]. Although highly informative, these approaches entail inherent limitations. Concentrations of cocoa polyphenols tested in most in vitro and ex vivo studies exceed those achievable through dietary intake [20], also, according to recent studies, the (−)-epicatechin is rapidly conjugated and metabolites are the most abundant polyphenols in plasma [14, 20]. Then, considering that the active compounds in cocoa may not be the native polyphenols, but the conjugated forms, in vitro and ex vivo studies using native compounds may not resemble the in vivo process. These metabolites may show different distribution patterns within tissues and cells and exert distinct biological effects [20]. Regarding the use of animal models, it is important to consider that the metabolites profile observed in experimental species differs widely from human [21].

Well-designed human studies have addressed the effects of cocoa consumption on clinical phenotypes but only a few have evaluated the underlying mechanisms. While contradictory results have been found, most studies suggests modulated expression of inflammatory cytokines, C-reactive protein and soluble adhesion molecules [22] after cocoa intake. The effect of other polyphenols on gene expression in human circulating cells has been analyzed [23], showing effects on inflammation and cell adhesion molecules [24, 25], however, the effect of cocoa polyphenols has not been studied. Thus, we performed a double-blinded, randomized, crossover clinical trial in healthy young adults to investigate the effects of high-polyphenols cocoa intake on gene expression and related signaling pathways in PBMCs, and to analyze the (-)-epicatechin metabolites profile, which may be related with an antioxidant capacity in plasma. Results will contribute to gain insight on the underlying signaling pathways of cocoa compounds in PBMCs.

## Methods

### Study design

**Fig. 1:**
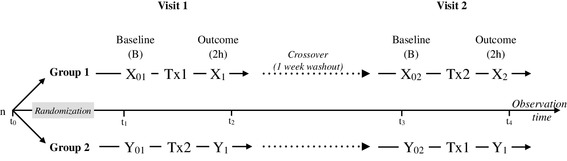
Study design. Double blinded, randomized, placebo-controlled, crossover clinical trial. Participants allocated in Group-1 consumed the treatments in Cocoa-Placebo sequence and participants allocated in Group-2 in Placebo-Cocoa order. Four blood samples were taken from each participant during the study; two samples at baseline state (t_1_ and t_3_), and two samples 2 h after treatment intake (t_2_ and t_4_). *Tx1* Cocoa, *Tx2* Placebo

A double blinded, randomized, placebo-controlled, crossover design (Fig. 1) was conducted in healthy young adults according to the principles of Good Clinical Practice and with the approval of the Ethics Committee of the National Institute of Genomic Medicine in Mexico City and registered as a clinical trial at Federal Commission for the Protection against Sanitary Risk according to Federal Regulations (registration number: 133300CT190199). Each participant provided written informed consent prior to his/her inclusion in the study. The study was conducted at the Nutrition Clinic of the Universidad Iberoamericana in Mexico City. Informed consent was obtained from all participants included in the study.

### Subjects

Eligible participants fulfilled the following inclusion criteria: age between 18 and 40 years, body mass index (BMI) between 18.5 and 30 kg/m^2^, non-smokers, and classified as sedentary to moderately active according to the International Physical Activity Questionnaire (IPAQ) [26]. Consumption of supplements, antioxidants or medication one month before and during the study was an exclusion criteria. Diet was not modified at any time but participants were asked to avoid polyphenol-rich products such as red wine, green tea, black tea, cocoa and cocoa-derived products at least 24 h before each visit. Subjects included in the gene expression analysis by microarray and real time qPRCR had a complete set of samples (*n* = 4; two treatments, under fasting conditions and 2 h after treatment) with sufficient amount (> 1 µg for microarray, > 500 ng for qPCR) and high RNA integrity number (RIN > 9).

### Intervention and test product

The intervention consisted of single dose (2 h) exposures to high-polyphenol cocoa powder or maltodextrins as control (referred as placebo). The dose and time for data collection were defined according previous studies suggesting a peak (−)-epicatechin concentration in plasma 2 h after consumption of 390–746 mg/day of polyphenols, of which 60–203 mg/day were from (−)-epicatechin [21, 28–30]. The cocoa pills contained ~ 1.3 g of a commercially available extract obtained from cocoa nibs (cocoa powder), dark red to red-violet in color and partially soluble in water which contained ~ 50% of polyphenols [~ 94 mg (−)-epicatechin], alkaloids (caffeine and theobromine), minerals and other cocoa compounds (Table 1). Cocoa nibs were unfermented, non-roasted, and blanch-treated to keep the maximum flavanol content [31]. The placebo pills contained ~ 1.3 g of a partially hydrolyzed polysaccharide (maltodextrins). Both products were encapsulated and delivered in pills of equal appearance. For this, participants were randomly allocated in Group-1 (Cocoa-Placebo) or Group-2 (Placebo-Cocoa) and appointed twice with one week washout period between the two visits to the clinic (Fig. 1). During the exposure, participants were not allowed to eat or drink anything except water. Treatment codes remained blind until the end of the data analysis. Food consumption was evaluated using a 24 h recall and a validated food frequency questionnaire (SNUT) [32]. Anthropometric measures (weight, height and waist circumference) were conducted as described by Lohman et al. [33]. Body composition (fat mass, fat-free mass, body water) analyses were also carried out at baseline using bioelectric impedance an In Body 250 equipment. Blood samples were drawn under fasting conditions and 2 h after treatment intake. Food consumption was not allowed in between blood collections. PBMCs were isolated from 8 mL of blood using Cell Preparation Tubes (CPT) containing sodium citrate (Cat. 362761, BD, Franklin Lakes, USA) (Vacutainer). Plasma was collected in 6 mL lithium heparin tubes (BD, Ref. 367884, Franklin Lakes, USA) for measurement of the antioxidant capacity of plasma and the concentration of (−)-epicatechin metabolites. The main determinations were the concentration levels of (−)-epicatechin metabolites in plasma, the antioxidant activity of plasma, and gene expression of PBMCs.

**Table 1:** Characterization of cocoa powder

Compound	mg/g
Total polyphenols contenta	500
Epicatechin	78
Flavanolsb	200
Flavan3-olsc	100
Theobromine	50

Values are mg/g of cocoa powder

^a^Determinations by Folin–Ciocalteu as catechin

^b^Determinations as catechin

^c^Determinations as catechin, epicatechin, B1 and B2

### Quantification of (−)-epicatechin metabolites in plasma samples

(−)-Epicatechin metabolites were identified and quantified in plasma samples taken from healthy young adults by ultra-high performance liquid chromatography tandem–mass-spectrometry (UHPLC–MS/MS) as described by Actis-Goretta et al. [11]. Briefly, plasma was obtained by whole blood centrifugation (heparin-containing vacutainers) (BD, Ref. 367884, Franklin Lakes, USA) at 1500×*g* for 10 min at 10 °C, and spiked with stabilization buffer (ascorbic acid 1.136 M, EDTA 3.43 mM, pH 3.6) at a final ratio of 20 µL of buffer per 1 mL of collected plasma. Plasma was snap-frozen in liquid nitrogen and stored at − 80 °C until quantification of (−)-epicatechin metabolites. Plasma was subsequently thawed on ice for three hours and homogenized. Proteins and phospholipids were removed from plasma samples by transferring 200 µL of plasma into a 96-wells Ostro plate from Waters (Ref. 186005518, Baden, Switzerland) and mixing it with 600 µL of acetonitrile, to finally filtrate the plasma during 5 min using a vacuum manifold from Waters (Ref. 186001831, Switzerland). The filtered sample was collected in a second 96-wells plate (Waters, Ref. WAT058957, Baden, Switzerland) and 200 µL of methanol were added to complete the cleaning procedure. Then, filtered samples were dried using nitrogen at room temperature and stored at − 20 °C until analysis. The dried residue was resuspended in 100 µL of 8% acetonitrile in acidic water and 5 µL was injected into the UHPLC–MS/MS system (Waters Acquity hyphenated to an AB Sciex QTRAP5500, operating in electrospray negative ionization mode) for detection and quantification of (−)-epicatechin metabolites. Quantification of (−)-epicatechin metabolites was performed by applying matrix-matched calibration curves built with eight standards concentrations (10, 20, 50, 100, 200, 500, 1000, 2000 nM). Internal standards (umbelliferone sulfate, umbelliferone glucuronide and (+)-4′-*O*-methylcatechin) were spiked in plasma samples at the beginning of the sample preparation for accurate quantification. (−)-Epicatechin metabolites and internal standards were a gift of Dr. Lucas Actis Goretta, of the Nestlé Research Center (NRC, Lausanne, Switzerland). (−)-Epicatechin (EC), (+)-catechin (cat), (−)-epicatechin-3′-*O*-glucuronide (E3′G), (−)-epicatechin-4′-*O*-glucuronide (E4′G), (+)-catechin-4′-*O*-glucuronide (C4′G), (−)-epicatechin-3′-*O*-sulfate (E3’S), (−)-epicatechin-4′-*O*-sulfate (E4’S), (−)-3′-O-methylepicatechin-4′-sulfate (3′ME4S), (−)-4′-*O*-methylepicatechin (4′ME) and (−)-3′-*O*-methylepicatechin (3′ME) were identified and quantified. All samples were processed in duplicate and analyzed using Analyst Software, version 1.6.

### Quantification of total antioxidant capacity of plasma

Plasma samples were analyzed using the Antioxidant Assay Kit from Sigma-Aldrich (St. Louis, USA) according to the manufacturer’s protocol and total antioxidant activity of plasma was calculated by calibration curves built with six standards concentrations (0, 0.015, 0.045, 0.105, 0.21, 0.42 mM) and using Trolox™ (water-soluble vitamin E analog) as the standard or control antioxidant. All samples were analyzed in duplicate.

### RNA Preparation

Total RNA was isolated from PBMCs using the TRIzol Reagent according to the manufacturer’s instructions (Ambion, Carlsbad, USA). The isolated RNA was eluted in 40 µl of RNase/free water and its concentration and integrity were assessed by electrophoresis using the 2100 Bioanalyzer System (Agilent Technologies, Palo Alto, CA, USA). An RNA integrity number (RIN) > 9 was established as acceptable for microarray analysis.

### Microarray processing

The transcriptomic profile of PBMCs was evaluated by array technology using the HumanV4GEX Expression BeadChip from Illumina. Processing of RNA samples was performed according Illumina protocols using the TotalPrep™ RNA amplification kit (Illumina, Carlsbad, USA) to generate biotinylated antisense RNA (aRNA) copies of each mRNA in a sample. The last step in RNA amplification was the purification of aRNA to eliminate unwanted compounds. Afterwards, aRNA was used in microarray to measure gene expression.

### Gene expression profiling

Gene expression profiling was performed on a subset of participants that met the microarray criteria of RNA quality and concentration. The treatment’s effect on transcriptomic of PBMCs was evaluated using baseline and 2 h cell collection after both treatments. Low quality samples (RIN < 9) were eliminated from microarray analysis as they can affect the gene expression results by carry-over of contaminating factors, salts, alcohols, and phenol [27]. All possible pairwise comparisons between treatments and conditions (fasting vs 2 h) were analyzed. However, to evaluate the effects of treatment’s intake, results presented here focused on comparing the list of differentially expressed genes resulting from contrasting 2 h vs baseline of samples treated with cocoa (Supplementary Fig. 1).

### Microarray data analysis

Raw data were background-corrected using Robust Multiarray Average (RMA) [28] and normalized using Quantile Normalization [29]. Differential expression was determined using statistical linear models with arbitrary coefficients, contrasts of interest were analyzed using the bioconductor library Limma [30, 31]. Benjamini and Hochberg multiple testing correction known as false discovery rate (FDR) [32] was applied to correct for multiple tests and to control the number of false positives. Genes were selected as differentially expressed based on *p* value < 0.05. An implementation of multiscale bootstrap resampling was performing using the package “pvclust” for assessing the uncertainty in hierarchical cluster analysis [33].

### Functional enrichment analysis

Functional enrichment analysis was implemented with IPA (QIAGEN Redwood City, CA, USA). IPA assesses enrichment using a Fisher exact *p* value. Additionally, it computes a *Z* score that allows inferring upstream transcriptional regulators and expectable enriched functions, based on statistical significance by comparing the match between observed and predicted up/down regulation patterns. Predicted regulation patterns are based on previously reported causal relationships between relevant genes and functions [34].

### cDNA synthesis and real-time PCR

The expression level of five differentially expressed genes [Interleukin 8 Receptor, Beta (*CXCR2 referred as IL8RB)*, Adrenoceptor Beta 2 *(ADRB2)*, Formyl Peptide Receptor 1 (*FPR1), CD36* and Interleukin 8 *(CXCL8 referred as IL8*)] was confirmed in a different subset of participants by real-time qPCR using the Quantum DNA analyzer (Applied Biosystems). Up-regulated (*IL8RB, ADRB2*) and down-regulated (*FPR1, CD36, IL8*) genes were selected based in their fold-change and relevance for the study project. cDNA was synthesized from 500 ng of total RNA using an oligo-dT primer and M-MuLV reverse transcriptase (#K1216, Thermo Scientific) according the instructions of the manufacturer. A volume of 2 µL of cDNA was PCR amplified using Taqman probes (Applied Biosystems) in a 10 µL reaction volume. The PCR products were quantified using standard curves built with six standards concentrations (0.32, 1.6, 6, 40, 200, 1000 nM). Data analysis was performed according to the absolute quantification method.

### Statistical analysis

Sample size of the study was calculated based on a previous study conducted at our laboratory that identified a moderate effect size (Cohen’s *d* = 0.7) on gene expression in PBMCs after a single dose of high polyphenols cocoa intake. Then, we required *n* = 20 subjects to complete the two treatments to have an 80% power and a confidence of *α* < 0.05. A descriptive analysis was conducted for study variables using SPSS v16 (Chicago, SPSS Inc.). Data are presented as mean and standard deviation. Carry-over effect between treatments was assessed by t-test for independent samples. Differences in plasma (−)-epicatechin metabolites, and plasma antioxidant activity at baseline and 2 h after cocoa and placebo intake were analyzed using a t-test for related samples. Statistic differences were established at *p* < 0.05. Microarray data analysis was performed using the statistical language R (3.3.2) (http://www.r-project.org/). Minimally detected effect size on gene expression was estimated by calculation of Cohen´s d using the difference in the expression of *IL8* after cocoa intake [35]. Post-hoc power analysis for gene expression differences was performed using the program G*Power [36].

## Results

### Study participants

**Fig. 2:**
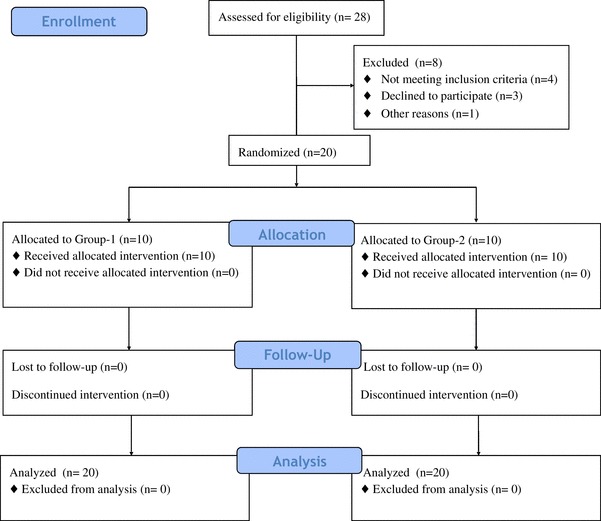
Flow diagram of participants during study. Participants allocated in Group-1 consumed treatments in a Cocoa-Placebo sequence and Group-2 in the opposite order. Treatments consisted in high-polyphenols cocoa intake {600 mg polyphenols of which 94 were (−)-epicatechin} and placebo (maltodextrins) pills

The study was advertised using posters and flyers. Interested volunteers were screened for eligibility according inclusion criteria (*n* = 28). Twenty healthy young adults (9 women) were enrolled and successfully finished the crossover study (Fig. 2). Half of the volunteers consumed treatments in a cocoa-placebo treatments sequence, the other half did the opposite order. No adverse effects were reported in any group during the study. Data from descriptive variables stratified by sex are shown in Table 2. No carryover effect between visits was found (*p* > 0.05).

**Table 2:** Descriptive data of participants

	Sex	Mean (SD)	Sig.	95% CI	
				Lower bound	Higher bound
Age (years)	F	28 (4.15)	0.92	− 3.96	4.32
	M	28 (4.57)			
Weight (kg)	F	57 (5.25)	0.00*	− 19.10	− 5.59
	M	69 (8.36)			
Height (cm)	F	163 (5.78)	0.01*	− 15.38	− 2.77
	M	172 (7.36)			
BMI (kg/m^2^)	F	21 (1.6)	0.46	− 3.91	− 0.03
	M	23 (2.3)			

*F* female (*n* = 9), *M* male (*n* = 11), *BMI* body mass index

*n* = 20, *p < 0.05

### (−)-Epicatechin metabolites profiling

**Fig. 3:**
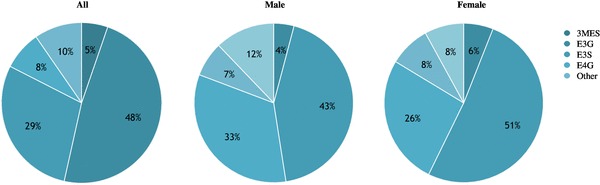
(−)-Epicatechin metabolites profiling after single dose cocoa intake stratified by sex. Ten metabolites were identified and quantified in plasma samples using UHPLC-MS/MS. Only seven metabolites increased their concentration levels after intervention (*p* < 0.05). Data presented as proportions in all (*n* = 20; 1474 ± 215 nM), male (*n* = 11; 1216 ± 295 nM) and female individual (n = 9; 1701 ± 305 nM). *3MES* (−)-3′-*O*-methylepicatechin-4′-sulfate, *E3G* (−)-epicatechin-3′-*O*-glucuronide, *E3S* (−)-epicatechin-3′-*O*-sulfate; *E4G* (−)-epicatechin-4′-*O*-glucuronide, *other E4’S* (−)-epicatechin-4′-O-sulfate, *EC* (−)-epicatechin, *C4′G* (+)-catechin-4′-*O*-glucuronide

(−)-Epicatechin metabolites from cocoa powder were identified and quantified in plasma samples by UHPLC–MS/MS. The concentration of (−)-epicatechin metabolites was under detection limits at baseline and increased to ~ 1500 nM after cocoa but not after placebo intake (*p* < 0.01). (−)-Epicatechin metabolites were classified according their conjunction group as *O-g*lucuronidated, *O-*Methylated and sulfated. The profile of (−)-epicatechin metabolites was not statistically different between treatment groups by sex groups (Fig. 3). E3G was the most abundant metabolite in 75% of subjects while the concentration of (+)-catechin, 4ME and 3ME were below of detection limit 2 h after cocoa intake.

### Change in antioxidant capacity of plasma

The effect of cocoa intake on the antioxidant capacity of plasma was evaluated by the Trolox equivalent antioxidant capacity (TEAC) method. In this study, we were unable to observe differences in the antioxidant activity in plasma after cocoa or placebo intake (Supplementary Fig. 2). Furthermore, the antioxidant capacity was not related to the concentration of any (−)-epicatechin metabolite or the sum of them.

### Effect of cocoa intake on gene expression

**Fig. 4:**
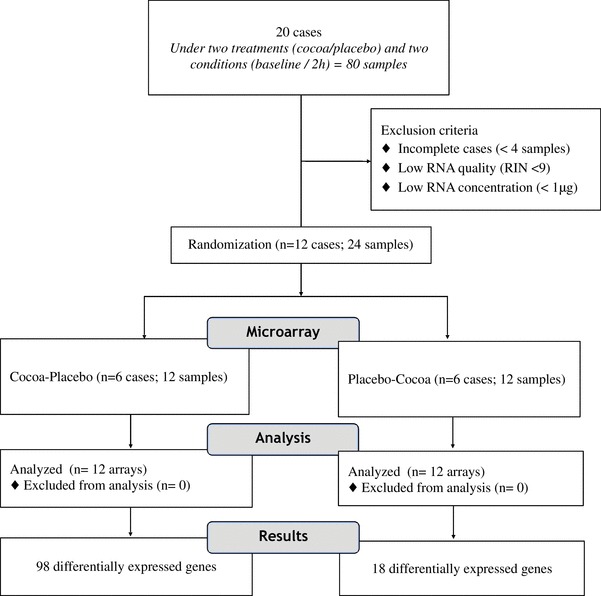
Flow diagram of RNA samples analyzed by microarray

**Fig. 5:**
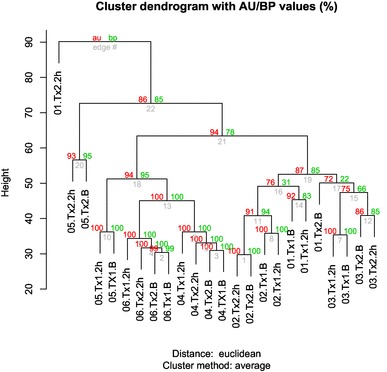
Cluster analysis of samples. Dendrogram of unsupervised hierarchical clustering on normalized gene expression values show a good agreement of samples according to their classification. Labels are defined as follows: subject ID {01:06}, treatment {Tx1: cocoa, Tx2: placebo} and condition {B: baseline and 2 h: after 2 h}. Clustering performed with Spearman correlation, Euclidean distance and average linkage. Numbers in red represents AU (approximately unbiased) *p* values (%). Numbers in green represents BP (bootstrap probability)

The single dose effect of cocoa and placebo intake on gene expression of PBMCs was analyzed in 24 RNA samples belonging to six participants (Fig. 4). Row data showed that most of the samples clustered by individual with approximately unbiased (AU) *p* values and a bootstrap probability (BP) value > 95 (Fig. 5). Data analysis demonstrated that the single dose ingestion of high-polyphenols cocoa pills induced changes in the expression levels of 98 genes of which 37 were down-regulated (Supplementary Table 1A), whereas the ingestion of placebo induced changes in the expression levels of 18 genes of which 3 were down-regulated (Supplementary Table 1B). The minimally detected effect size of *IL8* was moderate (Cohen’s *d* = 0.44) and the post-hoc power analysis considering gene expression data was 68% [35].

### Gene set enrichment analysis

**Fig. 6:**
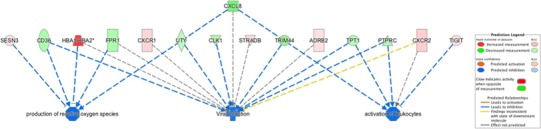
Enrichment analysis of differentially expressed genes after cocoa intake. Enrichment analysis was performed using the software Ingenuity Pathway Analysis (IPA). Three gene expression networks of annotated functions had an activation *z*-score < − 1.5 or > 1.5; production of ROS (*p* = 9.58E−4; *z*-score = − 2.216; activation of leukocytes (*p* = 4E−3; *z*-score = − 1.966); and, viral infection (*p* = 2.72E−2; *z* score = − 1.667)

To elucidate the role of the various genes for which the expression changed after cocoa and placebo intake, enrichment analysis of experimentally observed data using IPA were performed. The outputs of this analysis showed that differentially expressed genes after cocoa intake were related to 423 annotated functions but only 30 had an activation *z-score* value (Supplementary Table 2A). Among those, functions with an activation *z-*score < − 1.5 or > 1.5 were the following: (1) decreased production of reactive oxygen species (ROS) (*p* = 9.58E−4; *z*-score = − 2.216), (2) activation of leukocytes (*p* = 4E−3; *z-*score = − 1.966) and, (3) viral infection (*p* = 2.72E−2; *z*-score = − 1.667) (Fig. 6). Cellular configuration of the enriched pathways after cocoa intake is found in Supplementary Fig. 3. Other annotated functions which have been previously related to cocoa but had lower *z-*score were: both, Ca^2+^ quantity (*p* = 2.51E−05; *z*-score = 1.05) and mobilization (*p* = 3.62E−05; *z*-score = − 0.464). Differentially expressed genes after placebo intake were involved in 141 functions of these only two contained an activation *z-s*core value < 1 (Supplementary Table 2B). Changes in the expression levels of five differentially expressed genes (*IL8, IL8RB, CD36, ADRB2 and FPR1*) were validated using forty RNA samples belonging to ten participants by real time qPCR. Validated genes were selected according to their fold change and evidence of their participation in at least three functional pathways. Consistency in change direction between microarray and qPCR results was observed for *IL8, IL8RB, CD36* and *ADRB2*. Only *FPR1* showed differences in the change direction between the two methods (Table 3).

**Table 3:** cDNA synthesis and real-time PCR

	Log ratio		*p* value	
	Array	qPCR	Array	qPCR
CXCR2	↑ 0.869	↑ 0.061	6.13E−03	0.26
ADRB2	↑ 0.347	↑ 0.044	6.54E−03	0.05
FPR1	↓ − 0.396	↑ 0.035	8.07E−03	0.21
CD36	↓ − 0.396	↓ − 0.052	5.09E−03	0.04
CXCL8	↓ − 0.688	↓ − 0.295	7.36E−03	0.08

Differentially expressed genes validated by real-time PCR using the Quantum DNA analyzer (Applied Biosystems). Data analysis was performed according to the absolute quantification method

## Discussion

This study aimed to investigate the gene expression changes in PBMCs derived from a single-dose of high-polyphenols cocoa intake and control, and to analyze the circulating (−)-epicatechin metabolites, which may modulate the antioxidant capacity of plasma. Cocoa powder is a primary dietary source of (−)-epicatechin, commercially available and widely consumed. The (−)-epicatechin content may vary across products, cocoa powder used in the present study contains about four times more procyanidins and eight times more (−)-epicatechin than conventional cocoa. This dose is within the range of previous studies and albeit higher than the average population-based intake, it is considered as achievable by diet [37–39]. Confirmation of cocoa polyphenols bioavailability was performed by quantification of secondary metabolites in plasma samples 2 h after the intervention.

Differences in metabolites profile were observed between subjects, in agreement with previous findings [21, 40]. Inter-individual variation has been attributed to absorption, distribution, metabolism and excretion of bioactive compounds, as well as to heterogeneity in biological response of consumers [41, 42]. Sex differences in the expression of hepatic enzymes active in phase II metabolism have also been reported in human and animal models [43]. In the present study, females showed slightly higher concentration of circulating metabolites than males, nevertheless, the most abundant metabolites (E3G, E3S, E4G, 3MES) were similarly distributed and accounted for almost 85% of total concentration. Few studies have analyzed the metabolites profile in plasma, using different doses, vehicles and analytical methods [11, 21, 40, 44]. Results of our study show a similar concentration increase and distribution pattern than previous studies, although the lack of a standard for 3′-*O*-Methyl(−)-epicatechin-5-sulfate did not allow to quantify this metabolite [11]. The proportion of (−)-epicatechin glucuronides in this study, resemble findings by Rodriguez-Mateos et al. after consumption of a similar dose of (−)-epicatechin and are slightly higher than concentrations reported by Ottaviani et al. [21, 40]. As suggested by Actis-Goretta et al., the metabolites profile distribution might be influenced by the amount of ingested (−)-epicatechin; and the intake of a higher dose would result in higher relative concentrations of (−)-epicatechin glucuronides at expense of the conjugation of sulfates [11].

The antioxidant activity of cocoa and other flavonoid-rich products has been controversial since different results have been reported across studies [45]. The present study did not find significant changes in antioxidant capacity in plasma after cocoa intake, as previously reported [46, 47]. Actually, the increase of none of the analyzed (−)-epicatechin metabolites, or the sum of these, was related to changes in the antioxidant capacity of plasma. Recent findings by Ottaviani et al., reported the absence of oxidation products of (−)-epicatechin (ortho-quinones or quinone-related adducts) after consumption of radiolabeled (−)-epicatechin in human [21]. In addition, the low concentration of circulating metabolites after oral intake, makes unlikely that the biological effects mediated by (−)-epicatechin could be explained by a direct antioxidant mechanism. Thus, flavonoids may not act as antioxidant molecules, although some studies have identified that they modulate the antioxidant response by targeting different intracellular signaling pathways [45, 48, 49]. This response is coupled with the regulation of inflammation-related genes.

The effect of cocoa polyphenols on gene expression has been widely studied in in vitro and animal models. No reports from human interventional trials evaluating the effect of a single dose of cocoa or other catechin-rich products (green tea, red wine, dark chocolate) on gene expression were found. Findings of the present study are discussed in the context of well-performed clinical trials that assessed the effect similar flavonoids from olive oil, grape seeds and a mixed flavonoid-fish oil on subjects with different phenotypes (i.e., smokers, hypertensive, obese), during longer periods of time [50–53]. The exposure to flavanols from grape seeds (200 mg) triggered changes in 864 genes of leukocytes from seven male smokers, the observed changes are suggestive of lower immune cell adhesion to endothelial cells [51]. A study by Tome-Carneiro et al., conducted in type 2 diabetes and hypertensive patients with coronary artery disease, found that consumption of a grape extract fortified with resveratrol (8 mg) for one year down-regulated key inflammatory molecules [Tumor Necrosis Factor-alpha (*TNF-α*), Interleukin 1 Beta (*IL-1b*), *IL8*], in PBMCs [50]. Similar signaling pathways were modulated (antiviral and inflammation response) in whole-blood cells from obese women after consumption of a mixed flavonoid–fish oil containing epigallocatechin from green tea, quercetin, isoquercetin and omega-3 polyunsaturated fatty acid during 10 weeks [52]. Finally, consumption of olive oil during three weeks down-regulated the expression of genes participating in the renin–angiotensin–aldosterone system [Interleukin 8 Receptor, Alpha *IL8RA or CXCR1, ADRB2*, Angiotensin I Converting Enzyme (*ACE), NR1H2*] in PBMCs from healthy subjects [53]. The observed response in the present study is concordant with the mentioned findings, since similar transcripts (*IL8, IL8RA, ADRB2* and *FPR1)* and networks (decreased production of ROS, activation of leukocytes and viral response) were identified even after the intake of a single dose of high-polyphenols cocoa. Despite differences in time of exposure (single dose *vs* repeated doses over weeks or months), participant health status and type of flavonoids, results of the mentioned studies converged in a cell response characterized by an anti-inflammatory effects and decrease of production of ROS. This response attenuates the activation and migration of immune cells to endothelium, which may be associated to the immunological and cardiovascular benefits underlying cocoa intake [54]. In addition, a pathway associated with reduced viral infection has been identified in this and previous studies analyzing the effects of flavonoid on gene expression in blood cells [52], this effect could be related to the increase in factors that interfere with the ability of viruses to effectively infect and replicate within cells after flavonoids intake [52].

In this study, between- and within-subject variability was observed in gene expression. Thus, while the lists of differentially expressed genes after cocoa intake were not identical between Group-1 and Group-2, both suggest the same annotated pathways. Variation has been traced to difference in the relative proportions of cell-types, sex and age [42, 55]. However, it is unlike to have relevant fluctuations in cell-type frequencies after a single dose intervention [52, 56]. Transcriptional differences are more likely dependent on the baseline expression profile of participants, as suggested by the clustering of most of the samples from each individual as nearest neighbor. Differences in gene expression might also reflect the influence of genetic or environmental factors [55]. As recently proposed by Manach et al., a major challenge in nutrigenomics will be to develop methods and tools to phenotype and stratify individuals based on their ability to respond to plant food bioactive intake [42]. In conjunction, differentially expressed genes in this study merge in three major regulatory networks: (1) decreased ROS production [*FPR1, IL8*, Sestrin 3 (*SESN3*), *CD36*, Hemoglobin Subunit Alpha 1/2 (*HBA1*/*HBA2*)], (2) Ca^2+^ modulation [(*ADRB2, IL8, IL8RA, IL8RB, FPR1*, Protein Tyrosine Phosphatase, Receptor Type C (*PTPRC), TPT1 HBA1*/ *HBA2, ORM1*)], and (3) inflammatory response modulation (*IL8, PTPRC, TIGIT, TPT1, FPR1, IL8RA* and *IL8RB*). Calcium-mediated ROS modulation by (−)-epicatechin metabolites appears to play a prominent membrane-dependent mechanism by which these substances could exert biological effects [14, 48]. Other flavonoids have also been related to this effect in *in vitro* and animal models [57, 58].

The present study has several limitations. First, gene expression analysis was performed in a subgroup of participants with a full set of samples that fulfilled the requirements for microarray analysis. Despite the sample size and insufficient power (68%), the identified changes in gene expression are in agreement with findings of previous studies that tested the effect of other polyphenols in similar or larger samples [50–52]. The gene expression profile after each treatment suggests clearly different effects and validation of the results using real-time qPCR confirmed these findings. A second limitation of the study is that gene expression analysis was performed in PBMCs which include functional subgroups of cells, and do not necessarily reflect the effect of (−)-epicatechin metabolites in other tissues. Nevertheless, these cells have been extensively used to investigate the effects of nutritional interventions [59]. A third limitation is the use of a cocoa extract which contains other compounds besides polyphenols. While previous evidence suggests that (−)-epicatechin is the cause of the observed effects, cocoa extract contains a wide range of polyphenols and other bioactive compounds such as theobromine, and we cannot dismiss the contribution of these compounds to the observed effects. As strengths of this study, the crossover design allowed to control for confounder variables such as genetic background and the physiological characteristic of the study subjects, it also allowed to analyze the individual response under two conditions which increases the reliability of the results and optimize the sample size [60]. The simultaneous analysis of the conjugated metabolites and antioxidant capacity of plasma provided information on the lack of association between these measurements. This study confirmed that changes in gene expression may occur after a single dose of polyphenols within a short period of time.

The characterization of individual responses to polyphenols intake requires further investigation since responders and non-responders have been identified in other studies [42]. Studies using larger sample sizes, testing the short- and long-term effects of polyphenols on gene expression regulation are strongly recommended.

## Conclusion

In summary, high-polyphenol cocoa intake increased the concentration of (−)-epicatechin-derived metabolites in plasma 2 h after consumption without showing changes in the antioxidant capacity of plasma in the tested sample. The transcriptional response observed in this study was characterized by a moderated differential expression of inflammatory-related genes converging in three major regulatory networks: (1) decreased ROS production, (2) Ca^2+^ modulation, and (3) inflammatory response modulation. These pathways have been associated with the biological effects of other polyphenols and may contribute to the known benefits of cocoa consumption.

## Electronic supplementary material

Below is the link to the electronic supplementary material.

Supplementary material 1 (PDF 200 KB)

Supplementary material 2 (PDF 72 KB)

Supplementary material 3 (PDF 276 KB)

Supplementary material 4 (PDF 81 KB)

Supplementary material 5 (PDF 61 KB)
