# Supplementary material for: Gene expression changes by high-polyphenols cocoa powder intake: a randomized crossover clinical study
Source: Eur J Nutr. 2018 Jun 8;58(5):1887–98. doi: 10.1007/s00394-018-1736-8 (PMC6647247; doi:10.1007/s00394-018-1736-8)
Supplement: Supplementary file 1 — Supplementary material 1 (PDF 200 KB) [file 394_2018_1736_MOESM1_ESM.pdf]

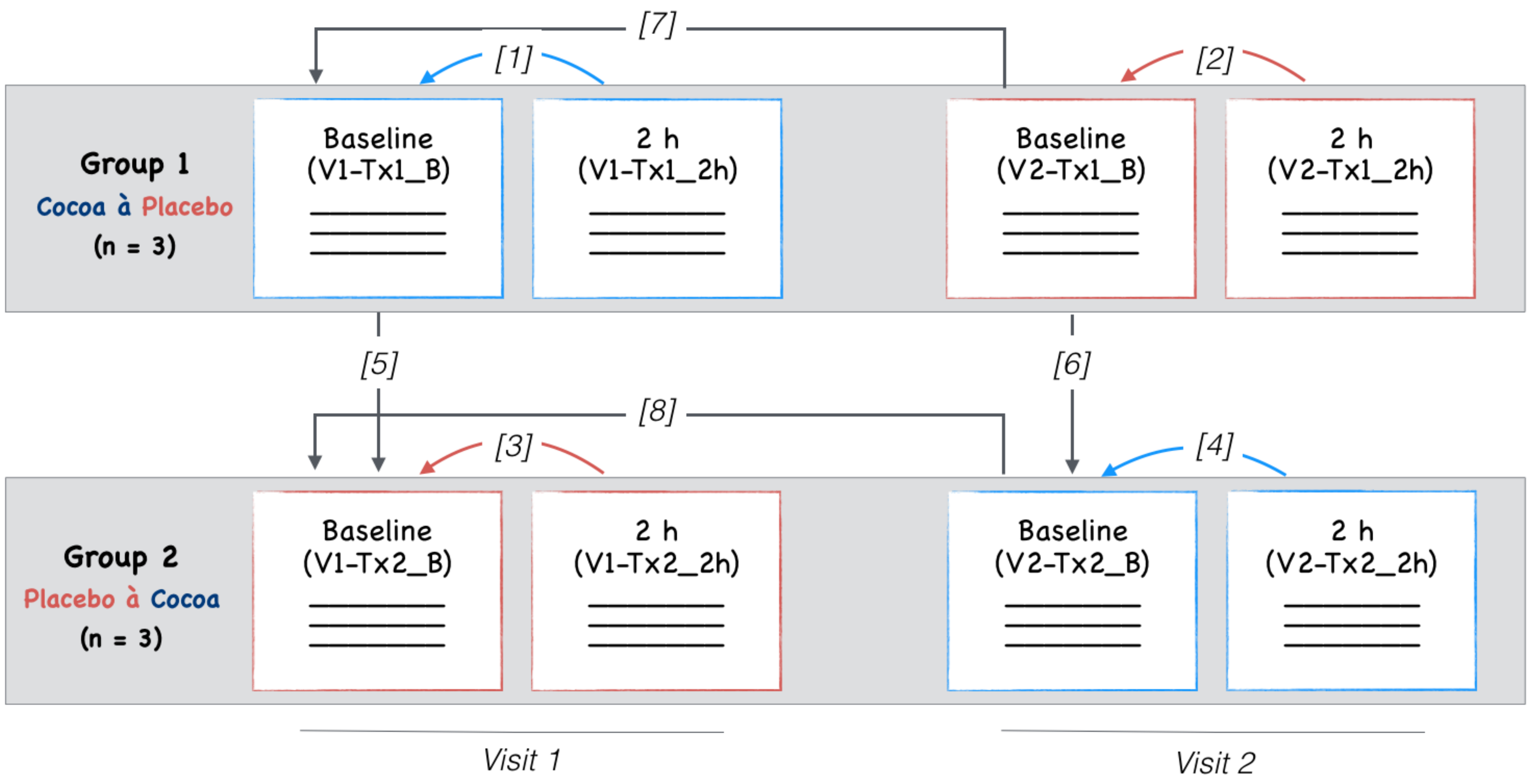

Supplementary Figure 1

Gene expression profiling was performed on RNA samples from a subset of randomly selected participants (n=6) to evaluate the treatment's effect at gene expression levels of the PBMCs. Samples included in the analysis were taken at baseline and 2 hours after cocoa and placebo intake. For analysis, RNA samples were classified per group. All possible pairwise contrast between groups were analyzed creating the contrasts of interest. Contrasts evaluated for this study are the acute response to cocoa {[1] and [4]}, andthe acute response to placebo {[2] and [3]}. Labels are defined as follows: V1:Visit-1; V2: Visit-2; Tx1: Cocoa; Tx2: Placebo; B:Baseline; 2h: after 2 hours

Gene Expression Changes by High-Polyphenols Cocoa Powder Intake. A Randomized Crossover Clinical Study.
European Journal of Nutrition

Barrera-Reyes PK[1], Hernández-Ramírez N[1], Cortés J[1], Poquet L[2], Meisser Redeuil K[2], Rangel-Escareño C[3], Kussmann M[4,], Silva-Zolezzi I[5], Tejero ME[1*]
[1] Nutrigenomics and Nutrigenetics, National Institute of Genomic Medicine, 14610 Mexico City, Mexico
[2] Vitamins & Phytonutrients, Nestlé Research Centre, CH-1000 Lausanne, Switzerland.
[3] Computational Genomics, National Institute of Genomic Medicine, 14610 Mexico City, Mexico.
[4] Systems Nutrition, Metabonomics and Proteomics, Nestlé Institute of Health Sciences, 1015 Lausanne, Switzerland; Current affiliation: Liggins Institute, 1142 Auckland, New Zealand.
[5] Metabolic Programming. Nestlé Research Centre, CH-1000 Lausanne, Switzerland.
* Correspondence: etejero@inmegen.gob.mx; Tel.: +52 (55) 5350-1145.
