# Supplementary material for: Gene expression changes by high-polyphenols cocoa powder intake: a randomized crossover clinical study
Source: Eur J Nutr. 2018 Jun 8;58(5):1887–98. doi: 10.1007/s00394-018-1736-8 (PMC6647247; doi:10.1007/s00394-018-1736-8)
Supplement: Supplementary file 2 — Supplementary material 2 (PDF 72 KB) [file 394_2018_1736_MOESM2_ESM.pdf]

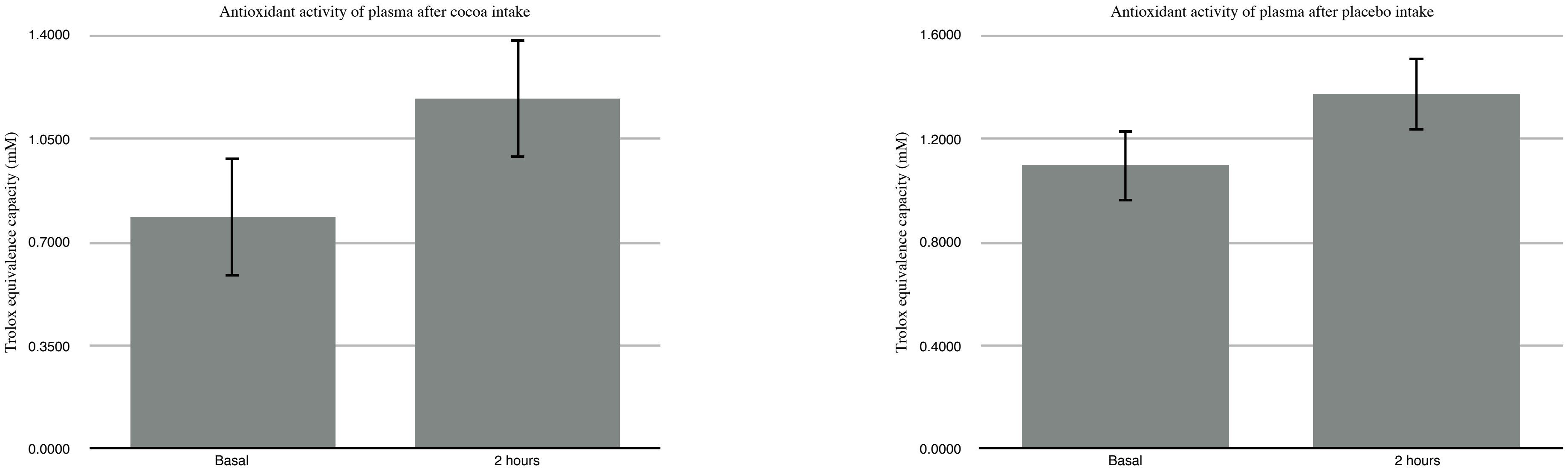

Supplementary Fig. 2: The presence of (-)-epicatechin metabolites in plasma is not related with antioxidant activity. *Total antioxidant activity was measured in plasma samples using a commercial kit. No differences were observed in basal state and 2 h after cocoa and placebo intake. Antioxidant activity of plasma was calculated applying external calibration curves. Data analyzed by t-test for related samples and expressed as mean and standard error in a sample of n=20*

Antioxidant-related Gene Expression Changes by High-Polyphenols Cocoa Powder Intake. A Randomized Crossover Clinical Study.

European Journal of Nutrition

Barrera PK[1], Hernández-Ramírez N[1], Cortés J[1], Poquet L[2], Meisser Redeuil K[2], Rangel-Escareño C[3], Kussmann M[4,], Silva-Zolezzi I[5], Tejero ME[1*]

[1] Nutrigenomics and Nutrigenetics, National Institute of Genomic Medicine, 14610 Mexico City, Mexico

[2] Vitamins & Phytonutrients, Nestlé Research Centre, CH-1000 Lausanne, Switzerland.

[3] Computational Genomics, National Institute of Genomic Medicine, 14610 Mexico City, Mexico.

[4] Systems Nutrition, Metabonomics and Proteomics, Nestlé Institute of Health Sciences, 1015 Lausanne, Switzerland; Current affiliation: Liggins Institute, 1142 Auckland, New Zealand.

[5] Metabolic Programming. Nestlé Research Centre, CH-1000 Lausanne, Switzerland.

* Correspondence: etejero@inmegen.gob.mx; Tel.: +52 (55) 5350-1145.
