# Supplementary material for: Gene expression changes by high-polyphenols cocoa powder intake: a randomized crossover clinical study
Source: Eur J Nutr. 2018 Jun 8;58(5):1887–98. doi: 10.1007/s00394-018-1736-8 (PMC6647247; doi:10.1007/s00394-018-1736-8)
Supplement: Supplementary file 4 — Supplementary material 4 (PDF 81 KB) [file 394_2018_1736_MOESM4_ESM.pdf]

Supplementary Table 1A Differentially expressed genes after cocoa intake

Log Ratio	p-value	ID	Symbol	Entrez Gene Name	Location	Type(s)
**0.33**	3.83E-03	FAM108C1	ABHD17C	abhydrolase domain containing 17C	Other	enzyme
**0.347**	6.54E-03	ADRB2	ADRB2	adrenoceptor beta 2	Plasma Membrane	G-protein coupled receptor
**1.885**	1.37E-06	AHSP	AHSP	alpha hemoglobin stabilizing protein	Cytoplasm	other
**3.182**	7.9E-07	ALAS2	ALAS2	5'-aminolevulinate synthase 2	Cytoplasm	enzyme
**-0.312**	5.99E-03	ANKRD54	ANKRD54	ankyrin repeat domain 54	Nucleus	transcription regulator
**0.359**	7.7E-03	ARHGAP17	ARHGAP17	Rho GTPase activating protein 17	Cytoplasm	other
**1.099**	6.95E-05	CA1	CA1	carbonic anhydrase 1	Cytoplasm	enzyme
**-0.508**	6.9E-03	CAPG	CAPG	capping actin protein, gelsolin like	Nucleus	other
**-0.396**	5.09E-03	CD36	CD36	CD36 molecule	Plasma Membrane	transmembrane receptor
**0.306**	7.88E-03	CETN1	CETN1	centrin 1	Nucleus	enzyme
**-0.319**	4.57E-03	CKAP4	CKAP4	cytoskeleton associated protein 4	Cytoplasm	other
**-0.41**	2.38E-03	CLK1	CLK1	CDC like kinase 1	Nucleus	kinase
**-0.455**	9.52E-03	COTL1	COTL1	coactosin like F-actin binding protein 1	Cytoplasm	other
**-0.688**	7.36E-03	IL8	CXCL8	C-X-C motif chemokine ligand 8	Extracellular Space	cytokine
**0.734**	6.84E-03	CXCR1	CXCR1	C-X-C motif chemokine receptor 1	Plasma Membrane	G-protein coupled receptor
**0.869**	6.13E-04	IL8RB	CXCR2	C-X-C motif chemokine receptor 2	Plasma Membrane	G-protein coupled receptor
**0.49**	2.77E-03	WDR40A	DCAF12	DDB1 and CUL4 associated factor 12	Cytoplasm	other
**0.401**	7.85E-03	DDR1	DDR1	discoidin domain receptor tyrosine kinase 1	Plasma Membrane	kinase
**1.214**	4.36E-06	EPB42	EPB42	erythrocyte membrane protein band 4.2	Plasma Membrane	transporter
**0.557**	8.72E-04	FAM46C	FAM46C	family with sequence similarity 46 member C	Extracellular Space	other
**-0.396**	8.07E-03	FPR1	FPR1	formyl peptide receptor 1	Plasma Membrane	G-protein coupled receptor
**0.316**	9.34E-03	GFI1	GFI1	growth factor independent 1 transcriptional repressor	Nucleus	transcription regulator
**0.436**	4.11E-03	GYPE	GYPE	glycophorin E (MNS blood group)	Plasma Membrane	other
**3.707**	2.24E-05	HBA1	HBA1/HBA2	hemoglobin subunit alpha 2	Extracellular Space	transporter
**1.729**	1.34E-03	HBA2	HBA1/HBA2	hemoglobin subunit alpha 2	Extracellular Space	transporter
**1.581**	5.9E-04	HBA2	HBA1/HBA2	hemoglobin subunit alpha 2	Extracellular Space	transporter
**2.202**	3.99E-06	HBD	HBD	hemoglobin subunit delta	Other	transporter
**2.137**	5.48E-05	HBE1	HBE1	hemoglobin subunit epsilon 1	Cytoplasm	transporter
**1.582**	3.43E-04	HBM	HBM	hemoglobin subunit mu	Cytoplasm	other
**0.73**	3.78E-03	IFIT1L	IFIT1B	interferon induced protein with tetratricopeptide repeats 1B	Cytoplasm	other
**-0.436**	2.08E-03	HS.552896	KCNC1	potassium voltage-gated channel subfamily C member 1	Plasma Membrane	ion channel
**0.748**	1.37E-03	KCNG1	KCNG1	potassium voltage-gated channel modifier subfamily G member 1	Plasma Membrane	ion channel
**-0.395**	3.67E-03	KREMEN2	KREMEN2	kringle containing transmembrane protein 2	Other	other
**0.323**	5.3E-03	KRTAP9-5	KRTAP9-9	keratin associated protein 9-9	Cytoplasm	other
**0.396**	7.87E-03	LARGE	LARGE1	LARGE xylosyl- and glucuronyltransferase 1	Cytoplasm	enzyme
**0.798**	8.36E-03	LOC440313	LOC440313	protein enabled homolog	Other	other
**-0.405**	1.45E-03	LRRN3	LRRN3	leucine rich repeat neuronal 3	Extracellular Space	other
**-0.428**	2.39E-03	MGST1	MGST1	microsomal glutathione S-transferase 1	Cytoplasm	enzyme
**-0.42**	1.47E-03	MIIP	MIIP	migration and invasion inhibitory protein	Other	other
**-0.448**	3.68E-03	MPZL2	MPZL2	myelin protein zero like 2	Plasma Membrane	other
**-0.364**	9.08E-03	MS4A6A	MS4A6A	membrane spanning 4-domains A6A	Other	other
**0.805**	3.37E-04	MUC6	MUC6	mucin 6, oligomeric mucus/gel-forming	Extracellular Space	other
**0.422**	3.05E-03	MYO1B	MYO1B	myosin IB	Cytoplasm	enzyme
**0.338**	4.98E-03	NIPAL4	NIPAL4	NIPA like domain containing 4	Other	other
**0.311**	9.24E-03	NUAK2	NUAK2	NUAK family kinase 2	Other	kinase
**0.466**	2.38E-03	ORM1	ORM1	orosomucoid 1	Extracellular Space	other
**-0.398**	1.76E-03	OSBPL8	OSBPL8	oxysterol binding protein like 8	Plasma Membrane	transporter
**-0.506**	3.06E-03	PFDN6	PFDN6	prefoldin subunit 6	Cytoplasm	other
**0.432**	6.36E-03	PKHD1	PKHD1	PKHD1, fibrocystin/polyductin	Plasma Membrane	other
**0.407**	3.61E-03	PPAPDC1B	PLPP5	phospholipid phosphatase 5	Other	phosphatase
**-0.314**	4.55E-03	A26C3	POTEG (includes others)	POTE ankyrin domain family member G	Other	other
**-0.372**	7.35E-03	PTPRC	PTPRC	protein tyrosine phosphatase, receptor type C	Plasma Membrane	phosphatase
**0.46**	8.2E-03	RAP1GAP	RAP1GAP	RAP1 GTPase activating protein	Cytoplasm	other
**1.019**	1.22E-03	RNF213	RNF213	ring finger protein 213	Cytoplasm	enzyme
**-0.905**	9.09E-03	RNU1G2	RNU1-4	RNA, U1 small nuclear 4	Other	other
**-0.582**	6.15E-03	RPPH1	RPPH1	ribonuclease P RNA component H1	Other	other
**0.644**	2.31E-04	SELENBP1	SELENBP1	selenium binding protein 1	Cytoplasm	other
**1.161**	1.66E-03	SERPINA13	SERPINA13P	serpin family A member 13, pseudogene	Extracellular Space	other
**0.428**	3.99E-03	SESN3	SESN3	sestrin 3	Extracellular Space	other
**0.933**	6.73E-03	SLC25A37	SLC25A37	solute carrier family 25 member 37	Cytoplasm	transporter
**0.635**	3.67E-03	SLC25A39	SLC25A39	solute carrier family 25 member 39	Cytoplasm	other
**0.421**	7.25E-03	RUNDC2C	SNX29P2	sorting nexin 29 pseudogene 2	Other	other
**0.676**	4.15E-04	STRADB	STRADB	STE20-related kinase adaptor beta	Cytoplasm	kinase
**0.376**	7.26E-03	TGFBR3	TGFBR3	transforming growth factor beta receptor 3	Plasma Membrane	kinase
**0.35**	5.64E-03	TIGIT	TIGIT	T-cell immunoreceptor with Ig and ITIM domains	Plasma Membrane	other

**-0.32**	8.1E-03	TMEM165	TMEM165	transmembrane protein 165	Plasma Membrane	other
**0.714**	8.4E-03	GPR175	TPRA1	transmembrane protein adipocyte associated 1	Plasma Membrane	G-protein coupled receptor
**-0.35**	9.36E-03	TPT1	TPT1	tumor protein, translationally-controlled 1	Cytoplasm	other
**-0.541**	5.43E-03	TRIM44	TRIM44	tripartite motif containing 44	Cytoplasm	other
**0.512**	7.14E-03	TSPAN5	TSPAN5	tetraspanin 5	Plasma Membrane	other
**-0.389**	6.43E-03	UGCG	UGCG	UDP-glucose ceramide glucosyltransferase	Cytoplasm	enzyme
**-0.501**	6.5E-03	UIMC1	UIMC1	ubiquitin interaction motif containing 1	Nucleus	other
**-0.315**	5.18E-03	UTY	UTY	ubiquitously transcribed tetratricopeptide repeat containing, Y-linked	Nucleus	enzyme
**-0.734**	1.75E-03	ZADH2	ZADH2	zinc binding alcohol dehydrogenase domain containing 2	Cytoplasm	enzyme
**-1.054**	4.12E-04	ZNF573	ZNF573	zinc finger protein 573	Nucleus	other
**1.181**	1.94E-05	LOC100131164	*Not identified*			
**0.95**	9.81E-04	LOC644852	*Not identified*			
**0.652**	2.16E-03	LOC389599	*Not identified*			
**0.609**	1.97E-03	LOC283663	*Not identified*			
**-0.599**	8.25E-03	LOC100129882	*Not identified*			
**0.576**	3.12E-03	LOC100131391	*Not identified*			
**0.56**	8.3E-03	LOC642469	*Not identified*			
**-0.375**	3.57E-03	LOC399804	*Not identified*			
**-0.357**	8.91E-03	LOC645351	*Not identified*			
**0.348**	8.6E-03	LOC644012	*Not identified*			
**0.322**	8.73E-03	HS.527387	*Not identified*			
**0.315**	5.22E-03	C1ORF183	*Not identified*			
**0.315**	9.78E-03	LOC374491	*Not identified*			
**-0.311**	4.8E-03	HS.545536	*Not identified*			
**-0.306**	7.07E-03	HS.578383	*Not identified*			
**0.306**	4.19E-03	LOC391347	*Not identified*			
**-1.045**	4.37E-03	RNU1-5	*Not identified*			
**0.393**	1.92E-03	LOC649185	*Not identified*			
**0.351**	9.59E-03	LOC100132506	*Not identified*			
**0.343**	2.56E-03	C1ORF21	*Not identified*			
**0.331**	5.06E-03	LOC648879	*Not identified*			
**-0.304**	4.73E-03	LOC728505	*Not identified*			
**-0.303**	5.83E-03	LOC729992	*Not identified*			

Supplementary Table 1B Differentially expressed genes after placebo intake

Log Ratio	p-value	ID	Symbol	Entrez Gene Name	Location	Type(s)
**0.467**	7.9E-03	AP2B1	AP2B1	adaptor related protein complex 2 beta 1 subunit	Plasma Membrane	transporter
**0.343**	9.22E-03	CACNA2D3	CACNA2D3	calcium voltage-gated channel auxiliary subunit alpha2delta 3	Plasma Membrane	ion channel
**0.63**	2.53E-03	CTSZ	CTSZ	cathepsin Z	Cytoplasm	peptidase
**-0.364**	7.08E-03	CXCR4	CXCR4	C-X-C motif chemokine receptor 4	Plasma Membrane	G-protein coupled receptor
**0.374**	5.34E-03	DLG4	DLG4	discs large MAGUK scaffold protein 4	Plasma Membrane	kinase
**0.404**	8.73E-03	ERH	ERH	enhancer of rudimentary homolog (Drosophila)	Nucleus	other
**0.472**	7.26E-04	G6PD	G6PD	glucose-6-phosphate dehydrogenase	Cytoplasm	enzyme
**0.407**	5.77E-03	GTF2H4	GTF2H4	general transcription factor IIH subunit 4	Nucleus	transcription regulator
**0.391**	5.68E-03	MEPCE	MEPCE	methylphosphate capping enzyme	Other	enzyme
**-0.422**	6.13E-03	PRR13	PRR13	proline rich 13	Nucleus	other
**-0.318**	4.61E-03	RBM3	RBM3	RNA binding motif (RNP1, RRM) protein 3	Cytoplasm	other
**0.62**	6.15E-03	UBE2E1	UBE2E1	ubiquitin conjugating enzyme E2 E1	Cytoplasm	enzyme
**-0.455**	4.29E-03	LOC389049	*Not identified*			
**0.519**	5.21E-03	DKFZP781G0119	*Not identified*			
**-0.38**	7.43E-03	LOC643452	*Not identified*			
**0.312**	8.37E-03	LOC554208	*Not identified*			
**-0.31**	5.25E-03	LOC653257	*Not identified*			
**0.335**	3.94E-03	LOC100130884	*Not identified*			

Gene Expression Changes by High-Polyphenols Cocoa Powder Intake. A Randomized Crossover Clinical Study.

European Journal of Nutrition

Barrera-Reyes PK[1], Hernández-Ramírez N[1], Cortés J[1], Poquet L[2], Meisser Redeuil K[2], Rangel-Escareño C[3], Kussmann M[4,], Silva-Zolezzi I[5], Tejero ME[1*]

[1] Nutrigenomics and Nutrigenetics, National Institute of Genomic Medicine, 14610 Mexico City, Mexico
[2] Vitamins & Phytonutrients, Nestlé Research Centre, CH-1000 Lausanne, Switzerland.
[3] Computational Genomics, National Institute of Genomic Medicine, 14610 Mexico City, Mexico.
[4] Systems Nutrition, Metabonomics and Proteomics, Nestlé Institute of Health Sciences, 1015 Lausanne, Switzerland; Current affiliation: Liggins Institute, 1142 Auckland, New Zealand.
[5] Metabolic Programming. Nestlé Research Centre, CH-1000 Lausanne, Switzerland.
* Correspondence: etejero@inmegen.gob.mx; Tel.: +52 (55) 5350-1145.
