# Supplementary material for: Gene expression changes by high-polyphenols cocoa powder intake: a randomized crossover clinical study
Source: Eur J Nutr. 2018 Jun 8;58(5):1887–98. doi: 10.1007/s00394-018-1736-8 (PMC6647247; doi:10.1007/s00394-018-1736-8)
Supplement: Supplementary file 5 — Supplementary material 5 (PDF 61 KB) [file 394_2018_1736_MOESM5_ESM.pdf]

Supplementary Table 2A: Functional Enrichment Pathways Modulated After Cocoa Intake

Category	Functions Annotation	p-Value	Activation z-score	Molecules	# Molecules
Free Radical Scavenging	Production of reactive oxygen species	9.58E-04	-2.216	CD36,CXCL8,FPR1,HBA1/HBA2,SESN3	5
Cell-To-Cell Signaling and Interaction, Hematological System Development and Function, Immune Cell Trafficking, Inflammatory Response	Activation of leukocytes	4E-03	-1.966	CXCL8,CXCR2,PTPRC,TIGIT,TPT1	5
Infectious Diseases	Viral Infection	2.72E-02	-1.667	CD36,CLK1,CXCL8,CXCR1,CXCR2,FPR1,HBA1/HBA2,HBE1,PTPRC,STRADB,TPT1,TRIM44,UTY	13
Lipid Metabolism, Small Molecule Biochemistry	Synthesis of lipid	3.02E-02	-1.462	COTL1,CXCL8,FPR1,LARGE,UGCG	5
Cell-To-Cell Signaling and Interaction	Activation of cells	6.11E-03	-1.418	CXCL8,CXCR1,CXCR2,PTPRC,TIGIT,TPT1	6
Lipid Metabolism, Small Molecule Biochemistry	Fatty acid metabolism	6.83E-04	-1.004	CD36,COTL1,CXCL8,FPR1,LARGE,OSBPL8,UGCG	7
Cell Morphology	Cell spreading	3.57E-03	-0.958	CD36,CXCL8,CXCR2,RAP1GAP	4
Cellular Movement	Migration of cells	2.27E-02	-0.818	ADRB2,CD36,CXCL8,CXCR1,CXCR2,DDR1,FPR1,ORM1,RAP1GAP,TGFBR3,TPT1	11
Cell-To-Cell Signaling and Interaction	Binding of cells	2.57E-03	-0.806	CD36,CXCL8,CXCR1,CXCR2,ORM1,RAP1GAP	6
Cellular Movement, Immune Cell Trafficking	Leukocyte migration	1.61E-02	-0.563	CXCL8,CXCR1,CXCR2,DDR1,FPR1	5
Cell-To-Cell Signaling and Interaction	Binding of blood cells	1.53E-04	-0.515	CXCL8,CXCR1,CXCR2,ORM1,RAP1GAP	5
Cell Signaling, Molecular Transport, Vitamin and Mineral Metabolism	Mobilization of Ca2+	3.62E-05	-0.464	ADRB2,CXCL8,CXCR1,CXCR2,FPR1,PTPRC,TPT1	7
Inflammatory Response	Inflammatory response	1.28E-02	-0.299	CXCL8,CXCR1,CXCR2,FPR1,ORM1	5
Cellular Movement, Hematological System Development and Function, Immune Cell Trafficking, Inflammatory Response	Chemotaxis of mononuclear leukocytes	1.92E-03	-0.278	CXCL8,CXCR1,CXCR2,FPR1	4
Cell Death and Survival	Apoptosis of leukemia cell lines	3.63E-02	-0.277	GFI1,PTPRC,TPT1,UGCG	4
Cellular Movement	Cell movement of blood cells	3.66E-03	-0.259	CD36,CXCL8,CXCR1,CXCR2,DDR1,FPR1	6
Cellular Movement	Chemotaxis of cells	1.4E-02	-0.227	CD36,CXCL8,CXCR1,CXCR2,FPR1	5
Cell-To-Cell Signaling and Interaction, Hematological System Development and Function, Inflammatory Response	Binding of professional phagocytic cells	5.29E-05	-0.211	CXCL8,CXCR1,CXCR2,ORM1	4
Cell-To-Cell Signaling and Interaction	Adhesion of blood cells	2.23E-04	-0.2	ADRB2,CD36,CXCL8,CXCR1,CXCR2,ORM1	6
Cellular Movement, Hematological System Development and Function, Immune Cell Trafficking, Inflammatory Response	Chemotaxis of neutrophils	3.13E-04	-0.153	CXCL8,CXCR1,CXCR2,FPR1	4
Cell-To-Cell Signaling and Interaction	Binding of tumor cell lines	4.16E-03	-0.152	CD36,CXCR1,CXCR2,RAP1GAP	4
Cell-To-Cell Signaling and Interaction, Hematological System Development and Function, Immune Cell Trafficking	Adhesion of immune cells	6E-03	-0.121	CXCL8,CXCR1,CXCR2,ORM1	4
Cardiovascular System Development and Function, Cellular Movement	Chemotaxis of endothelial cell lines	1.57E-07	-0.102	CD36,CXCL8,CXCR1,CXCR2	4
Cellular Development, Hematological System Development and Function	Differentiation of blood cells	4.15E-02	0	CXCL8,CXCR1,CXCR2,PTPRC	4
Cellular Movement	Cell movement	2.08E-02	0.082	ADRB2,CD36,CXCL8,CXCR1,CXCR2,DDR1,FPR1,NUAK2,ORM1,RAP1GAP,TGFBR3,TPT1	12
Cellular Movement	Transmigration of cells	3.92E-04	0.343	CXCL8,CXCR2,FPR1,RAP1GAP	4
Cardiovascular System Development and Function, Cellular Movement	Movement of vascular endothelial cells	2.56E-03	0.528	CD36,CXCL8,CXCR2,ORM1	4
Cardiovascular System Development and Function, Cellular Movement	Migration of endothelial cells	1.62E-02	0.927	CXCL8,CXCR1,CXCR2,ORM1	4
Cardiovascular System Development and Function, Cellular Movement	Cell movement of endothelial cells	4.06E-03	0.987	CD36,CXCL8,CXCR1,CXCR2,ORM1	5
Cell Signaling, Molecular Transport, Vitamin and Mineral Metabolism	Quantity of Ca2+	2.51E-05	1.047	CXCL8,CXCR1,CXCR2,FPR1,HBA1/HBA2,ORM1,PTPRC	7

Supplementary Table 2B: Functional Enrichment Pathways Modulated After Placebo Intake

Category	Functions Annotation	p-Value	Activation z-score	Molecules	# Molecules
Infectious Diseases	Infection by RNA virus	4.58E-04	0.471	AP2B1,CACNA2D3,CTSZ,CXCR4,UBE2E1	5
Infectious Diseases	Infection of tumor cell lines	6.24E-04	0.594	AP2B1,CTSZ,CXCR4,UBE2E1	4

Gene Expression Changes by High-Polyphenols Cocoa Powder Intake. A Randomized Crossover Clinical Study.

European Journal of Nutrition

Barrera-Reyes PK[1], Hernández-Ramírez N[1], Cortés J[1], Poquet L[2], Meisser Redeuil K[2], Rangel-Escareño C[3], Kussmann M[4,], Silva-Zolezzi I[5], Tejero ME[1*]

[1] Nutrigenomics and Nutrigenetics, National Institute of Genomic Medicine, 14610 Mexico City, Mexico
[2] Vitamins & Phytonutrients, Nestlé Research Centre, CH-1000 Lausanne, Switzerland.
[3] Computational Genomics, National Institute of Genomic Medicine, 14610 Mexico City, Mexico.
[4] Systems Nutrition, Metabonomics and Proteomics, Nestlé Institute of Health Sciences, 1015 Lausanne, Switzerland; Current affiliation: Liggins Institute, 1142 Auckland, New Zealand.
[5] Metabolic Programming. Nestlé Research Centre, CH-1000 Lausanne, Switzerland.
* Correspondence: etejero@inmegen.gob.mx; Tel.: +52 (55) 5350-1145.
